# Vericiguat in Elderly Patients with Heart Failure and Reduced Ejection Fraction: Clinical Perspectives from Pathophysiology to Implementation

**DOI:** 10.3390/jcm15155918

**Published:** 2026-07-29

**Authors:** Carmelo Massimiliano Rao, Fabiana Lucà, Roberto Ceravolo, Michele Massimo Gulizia, Sandro Gelsomino, Nadia Ingianni, Marco Vatrano, Daniela Chiappetta, Tommaso Scarpino, Giovanna Geraci, Fabrizio Oliva, Federico Nardi, Massimo Grimaldi, Iris Parrini

**Affiliations:** 1 Cardiology Unit, Santa Maria Degli Ungheresi Hospital, Polistena, 89024 Reggio Calabria, Italy; 2 Cardiology Department, Grande Ospedale Metropolitano of Reggio Calabria, 89124 Reggio Calabria, Italy; fabiana.luca92@gmail.com; 3 Cardiology Unit, Giovanni Paolo II Hospital, 88046 Lamezia Terme, Italy; roberto_ceravolo@yahoo.it; 4 Cardiology Department, Garibaldi Nesima Hospital, 95122 Catania, Italy; michele.gulizia60@gmail.com; 5 Cardiothoracic Department, Maastricht University Hospital, 6229 Maastricht, The Netherlands; sandro.gelsomino@gmail.com; 6 Cardiologic District, Azienda Sanitaria Provinciale Trapani, 91016 Trapani, Italy; nadiaing@hotmail.it; 7 Cardiology Unit, “Pugliese” Hospital, “Renato Dulbecco”, 88100 Catanzaro, Italy; marco.vatrano1975@gmail.com (M.V.); tom.scarpino@virgilio.it (T.S.); 8 Cardiology Unit, “Santissima Annunziata” Hospital, 87100 Cosenza, Italy; daniela.chiappetta@email.it; 9 Cardiology Department, Sant’Antonio Abate Hospital, Erice, 91016 Trapani, Italy; giovannageraci@hotmail.com; 10 Cardiology Unit, ASST Grande Ospedale Metropolitano Niguarda, 20162 Milano, Italy; fabrizio.oliva@ospedaleniguarda.it; 11 Cardiology Department, Santo Spirito Hospital, Casale Monferrato, 15033 Alessandria, Italy; federico.nardi1@gmail.com; 12 Cardiology Department, General Regional Hospital “F. Miulli’’, Acquaviva delle Fonti, 70021 Bari, Italy; m.grimaldi@miulli.it; 13 Cardiology Unit, Koelliker Hospital, 10134 Torino, Italy; irisparrini@libero.it

**Keywords:** heart failure (HF), heart failure with reduced ejection fraction (HFrEF), vericiguat, soluble guanylate cyclase (sGC) stimulator, cyclic guanosine monophosphate (cGMP), elderly, frailty, guideline-directed medical therapy (GDMT)

## Abstract

Elderly patients with heart failure (HF) represent a rapidly growing and highly heterogeneous population marked by multimorbidity, frailty, and polypharmacy. These age-related features profoundly influence the course of HF and modify therapeutic response. Vericiguat, a novel oral soluble guanylate cyclase (sGC) stimulator, targets the impaired nitric oxide (NO)-sGC-cyclic guanosine monophosphate (cGMP) pathway, a central mechanism in vascular dysfunction and maladaptive remodelling. Clinical evidence, primarily from the VICTORIA trial and subsequent studies, supports vericiguat in selected patients with heart failure with reduced ejection fraction (HFrEF), particularly following recent worsening HF despite guideline-directed medical therapy (GDMT). Most of the available evidence derives from HFrEF populations, whereas studies in heart failure with preserved ejection fraction (HFpEF) have not demonstrated significant clinical benefit. In addition, dedicated evidence in frail older adults and in patients with geriatric syndromes remains limited. This review summarises current knowledge on the pharmacological rationale, pharmacokinetics, and pivotal clinical studies of vericiguat, highlighting quantitative results and their implications for clinical implementation in elderly patients with HFrEF and complex clinical profiles.

## 1. Introduction

Heart failure (HF) remains one of the leading causes of cardiovascular (CV) morbidity and mortality worldwide and represents a major public health challenge, particularly in ageing populations. The prevalence of HF increases substantially with advancing age, affecting nearly 10% of individuals older than 75 years and accounting for a disproportionate number of HF hospitalisations (HHF), healthcare expenditures, functional decline, and mortality [1,2]. As life expectancy continues to rise, elderly patients now constitute the largest and fastest-growing segment of the HF population encountered in daily clinical practice. Against this demographic background, the increasing complexity of HF has become particularly evident. Rather than representing a single clinical entity, HF encompasses patients with reduced (HFrEF), mildly reduced (HFmrEF), and preserved ejection fraction (HFpEF), each characterised by distinct pathophysiological mechanisms, clinical trajectories, and therapeutic responses [3]. This heterogeneity extends beyond left ventricular ejection fraction (LVEF), reflecting a broad spectrum of underlying etiologies, including ischemic heart disease, arterial hypertension (AH), valvular heart disease, cardiomyopathies, and infiltrative or inflammatory disorders [3]. Among these phenotypes, worsening HFrEF remains the clinical setting in which vericiguat has been most extensively investigated and therefore represents the primary focus of the present review, with particular attention to elderly and frail patients [4].

The clinical management of HF in older adults is frequently complicated by the coexistence of multiple chronic conditions, including chronic kidney disease (CKD), diabetes mellitus (DM), anaemia, cognitive impairment, and frailty. These factors contribute to a higher burden of symptoms, increased vulnerability to adverse events, and reduced tolerance to pharmacological interventions [5,6].

Moreover, the clinical complexity of elderly patients often limits the applicability of evidence derived from conventional randomised clinical trials (RCTs), in which frail individuals, very old patients, and those with significant multimorbidity remain underrepresented [7,8]. Beyond its diagnostic role, N-terminal pro-B-type natriuretic peptide (NT-proBNP) is a cornerstone biomarker for risk stratification and prognostic assessment in HF. Higher NT-proBNP concentrations consistently correlate with greater disease severity and an increased risk of HHF and CV death, making serial NT-proBNP assessment an integral component of contemporary HF management [9,10,11]. Although HF encompasses multiple clinical phenotypes, major advances in treatment have been most pronounced in HFrEF, where disease-modifying pharmacological therapies have substantially improved prognosis.

Over the last decade, the introduction of guideline-directed medical therapy (GDMT), including angiotensin receptor–neprilysin inhibitors (ARNI), beta-blockers (B-B) [12], mineralocorticoid receptor antagonists (MRA) and sodium–glucose cotransporter-2 inhibitors (SGLT2i) havesubstantially improved outcomes in patients with HFrEF [1,13,14]. Nevertheless, despite contemporary multidrug treatment strategies, a considerable proportion of patients continue to experience recurrent episodes of worsening HF, repeated HHF, and progressive functional deterioration [15].

Accordingly, attention has progressively shifted toward complementary therapeutic strategies targeting biological pathways that are not directly addressed by conventional neurohormonal inhibition [7].

Among these pathways, impairment of the nitric oxide (NO)–soluble guanylate cyclase (sGC)–cyclic guanosine monophosphate (cGMP) signalling cascade has emerged as a key contributor to endothelial dysfunction, vascular stiffness, inflammation, oxidative stress, and adverse myocardial remodelling in HF [7]. Vericiguat, an oral sGC stimulator, was developed to restore activity in this pathway by directly stimulating sGC and enhancing responsiveness to endogenous NO, thereby increasing intracellular cGMP availability despite the oxidative milieu characteristic of chronic HF [7].

The clinical relevance of this mechanism has been supported by the VICTORIA trial, which demonstrated a significant reduction in the composite endpoint of CV death or HHF among patients with worsening HFrEF receiving vericiguat in addition to standard therapy [16]. However, important questions remain regarding the implementation of this treatment in elderly and frail populations, in whom age-related physiological changes, polypharmacy, renal dysfunction, and functional impairment may influence both treatment selection and clinical outcomes [8]. Furthermore, emerging evidence from subgroup analyses, observational studies, and real-world registries is progressively expanding our understanding of vericiguat beyond the setting of the pivotal RCT. Collectively, these findings have expanded the potential role of vericiguat beyond the pivotal trial while underscoring the need for dedicated evidence in elderly and clinically complex patients, who remain underrepresented in RCTs. The present review aims to provide a comprehensive overview of the pathophysiological rationale, pharmacological characteristics, clinical evidence, and practical implementation of vericiguat in elderly patients with HF. Particular emphasis is placed on frailty, multimorbidity, polypharmacy, and real-world applicability, with the objective of supporting a more individualised and clinically relevant use of this therapy in contemporary CV practice.

## 2. Literature Search and Evidence Synthesis

### 2.1. Literature Search and Study Selection

This review was designed as a structured narrative review to summarise the available evidence on the efficacy, safety, tolerability, and clinical implementation of vericiguat in elderly patients with HF, with particular focus on frailty, multimorbidity, renal dysfunction, and polypharmacy. Although a structured literature search was performed and a PRISMA-style flow diagram is presented to improve transparency of study identification and selection, the methodology was not intended to meet the requirements of a formal systematic review and should not be interpreted as full PRISMA adherence. This review was designed as a structured narrative review rather than a formal systematic review or meta-analysis. The literature search and study selection process were performed to improve the transparency and reproducibility of the evidence synthesis. No formal risk-of-bias assessment, quality scoring system, or PRISMA checklist was undertaken, as these procedures were beyond the scope of the present narrative review.

A structured literature search was performed in PubMed/MEDLINE from database inception to 1 May 2026, to identify studies evaluating vericiguat in patients with HF. The search strategy combined Medical Subject Headings (MeSH) terms and free-text keywords related to HF, ageing, frailty, and vericiguat, including: “heart failure”, “HFrEF”, “HFpEF”, “elderly”, “older adults”, “geriatric”, “frailty”, “frail”, “octogenarian”, “nonagenarian”, and “vericiguat”. Additional manual screening of reference lists from relevant reviews and original investigations was also performed to identify potentially eligible studies not captured during the electronic search.

Titles and abstracts were independently screened by F.L. and C.M.R., and disagreements regarding study relevance were resolved through discussion with I.P. and R.C. Full-text assessments were subsequently performed to evaluate methodological relevance and applicability to elderly and clinically complex HF populations.

Studies were considered eligible if they fulfilled at least one of the following criteria:

1. RCTs evaluating vericiguat in HF;
2. Subgroup analyses focused on older adults or age-related outcomes;
3. observational or real-world studies involving elderly or multimorbid HF populations;
4. systematic reviews or meta-analyses investigating vericiguat efficacy and safety;
5. studies addressing frailty, polypharmacy, renal dysfunction, or geriatric management relevant to vericiguat use in HF.

Articles were excluded if they:

6. did not investigate vericiguat;
7. lacked clinically relevant HF outcomes;
8. focused exclusively on economic or pharmacoeconomic analyses without clinical data;
9. did not provide information applicable to elderly or clinically complex HF populations;
10. were editorials, conference abstracts without sufficient methodological detail, or duplicate publications.

**Figure 1:**
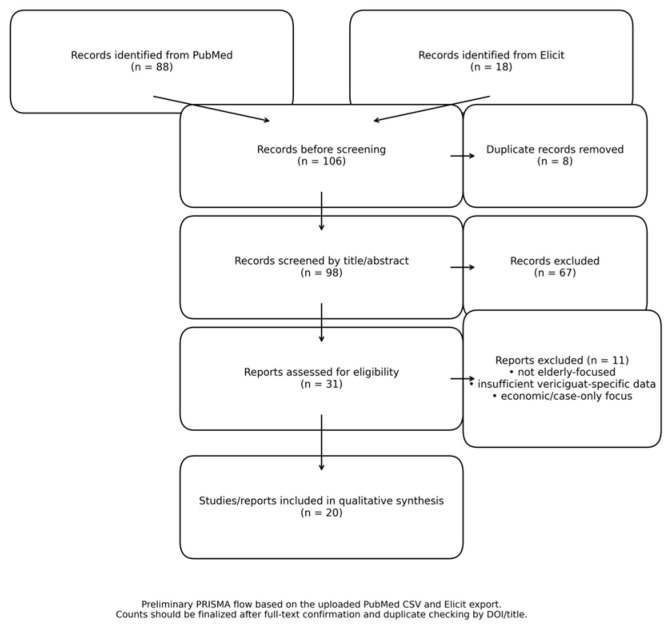
Supportive PRISMA-style flow diagram illustrating the literature identification and study selection process used for this structured narrative review. The diagram is presented to enhance transparency and reproducibility and does not imply a formal systematic review methodology or full PRISMA adherence.

The literature search initially identified 106 records. After removing duplicate publications and screening titles and abstracts, 20 studies and reports were included in the final qualitative synthesis. The process of study identification, screening, eligibility assessment, and final inclusion is illustrated in Figure 1. The principal characteristics of the studies included in the qualitative synthesis are summarised in Table 1. Because dedicated randomised clinical trials exclusively enrolling elderly patients with HF remain limited, the final evidence synthesis integrated data from RCTs, post hoc analyses, observational studies, real-world registries, and contemporary geriatric HF literature relevant to frailty, multimorbidity, and therapeutic complexity.

### 2.2. Data Extraction and Evidence Synthesis

Data extraction was independently performed by F.L. and C.M.R. and subsequently verified by I.P. and R.C. Extracted variables included study design, patient population, age distribution, HF phenotype, comorbidities, background pharmacological therapy, efficacy outcomes, safety endpoints, and tolerability findings. Particular attention was devoted to geriatric-specific aspects, including frailty-related variables, renal dysfunction, polypharmacy, symptomatic hypotension, syncope, anaemia, and treatment discontinuation.

Given the marked heterogeneity of the available evidence, including randomised clinical trials, observational cohorts, registries, narrative reviews, and meta-analyses, a quantitative pooled meta-analysis was not performed. Findings were therefore synthesised descriptively, emphasising clinically relevant aspects for elderly patients with HF, including implementation feasibility, multimorbidity burden, renal function, and integration with GDMT.

Priority was given to studies directly relevant to elderly or clinically complex HF populations, including subgroup analyses from the VICTORIA trial, real-world observational experiences, and investigations specifically addressing frailty, therapeutic burden, and geriatric management in patients treated with vericiguat. Given the narrative nature of this review, the findings should be interpreted as a qualitative synthesis of the currently available evidence rather than as a formal evidence-grading process. Consequently, no quantitative certainty assessment, risk-of-bias evaluation, or evidence-quality scoring was performed.

## 3. Pathophysiology

The NO-sGC-cGMP pathway plays a central role in CV homeostasis. Under physiological conditions, endothelial-derived NO diffuses into vascular smooth muscle cells and activates sGC, promoting intracellular cGMP synthesis. Increased cGMP levels subsequently activate protein kinase G, resulting in vasodilation, anti-fibrotic signalling, and improved myocardial relaxation [29,30]. In HF, oxidative stress and chronic inflammation reduce NO bioavailability and promote oxidation of the sGChaem moiety, thereby impairing enzyme responsiveness. Consequently, cGMP production declines, contributing to increased vascular resistance, myocardial hypertrophy, and diastolic dysfunction.

**Figure 2:**
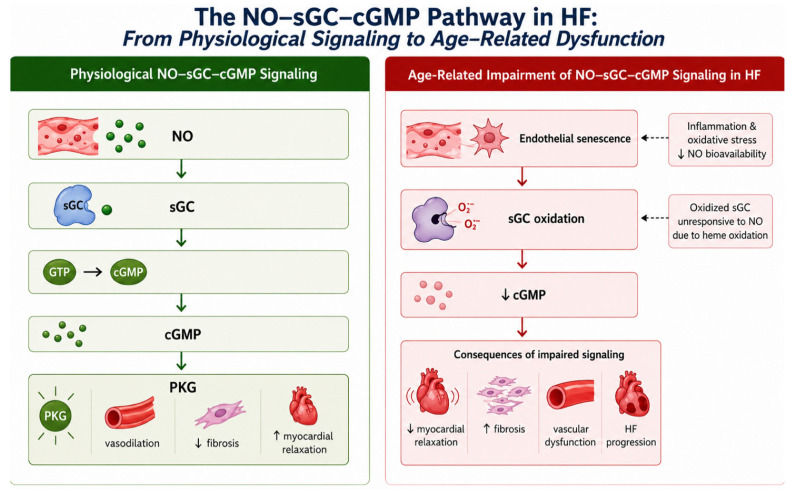
Age-related impairment of the NO–sGC–cGMP pathway in geriatric heart failure. Abbreviations: cGMP: cyclic guanosine monophosphate; GTP: guanosine triphosphate; HF: heart failure; NO: nitric oxide; PKG: protein kinase G; sGC: soluble guanylate cyclase.

**Figure 3:**
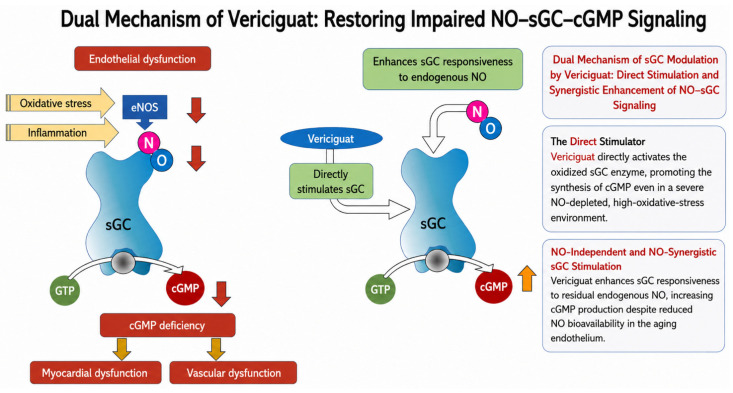
Proposed mechanisms of action of vericiguat on the NO–sGC–cGMP pathway. Abbreviations: cGMP: cyclic guanosine monophosphate; eNOS: endothelial nitric oxide synthase; GTP: guanosine triphosphate; HF: heart failure; NO: nitric oxide; sGC: soluble guanylate cyclase.

Ageing further amplifies impairment of the NO–sGC–cGMP pathway (Figure 2). Endothelial senescence and excessive reactive oxygen species blunt NO synthesis, while common age-related comorbidities, including AH, DM, and atherosclerosis, further impair sGC activation. These mechanisms may partly explain the high prevalence of both HFpEF and HFrEF in elderly populations. By restoring cGMP signalling, vericiguat may partially counteract these pathophysiological alterations by directly stimulating sGC and increasing its sensitivity to endogenous NO. However, the clinical relevance of these mechanisms in elderly patients has not yet been specifically established and remains largely inferred from mechanistic and clinical studies conducted in broader HFrEF populations [31] (Table 2). This dual mechanism may promote vasorelaxation and attenuate maladaptive remodelling even in the oxidative environment characteristic of the ageing myocardium (Figure 3).

Schematic representation of the physiological NO–sGC–cGMP signaling pathway and its age-related dysregulation in HF. Endothelial senescence, chronic inflammation, and oxidative stress are associated with reduced nitric oxide bioavailability and impaired soluble guanylate cyclase activation, which may contribute to decreased cGMP signalling, increased vascular stiffness, and progressive myocardial dysfunction. The figure illustrates the proposed pathophysiological mechanisms, based on current experimental and clinical evidence, and is intended as a conceptual representation rather than as direct evidence of clinical effects in elderly patients.

Vericiguat directly stimulates soluble guanylate cyclase and enhances its responsiveness to endogenous nitric oxide, thereby increasing cGMP production. These pharmacological effects are thought to contribute to improved vasodilatory signalling and to mechanisms associated with myocardial relaxation and attenuation of adverse cardiac remodelling in patients with HFrEF. The figure illustrates the proposed biological mechanisms underlying vericiguat and should not be interpreted as direct evidence of clinical benefit in elderly or frail patients.

## 4. Pharmacology and Pharmacokinetics in the Elderly

Vericiguat is administered orally once daily, exhibits high bioavailability, and demonstrates linear pharmacokinetics. Following administration with food, peak plasma concentrations are generally reached within 1–2 h, while steady-state levels are achieved after approximately four days. Vericiguat is primarily metabolised through hepatic glucuronidation via UGT1A9 and UGT1A1, whereas renal elimination of the parent compound is minimal [32]. Its elimination half-life of approximately 20 h supports convenient once-daily administration.

Population pharmacokinetic analyses have shown no clinically meaningful influence of age, sex, or mild-to-moderate renal impairment on vericiguat exposure. Consequently, dose adjustment is generally not required solely on the basis of age [32]. However, vericiguat has not been extensively evaluated in patients with severe hepatic dysfunction or estimated glomerular filtration rate (eGFR) < 15 mL/min/1.73 m^2^. Since ageing is frequently associated with reduced hepatic perfusion, altered body composition, and lower protein binding, cautious titration remains advisable in frail older adults.

Drug–drug interactions appear limited. Vericiguat neither inhibits nor induces major cytochrome P450 enzymes and shows low potential for clinically relevant P-glycoprotein interactions. Nevertheless, concomitant use with other sGC stimulators, such as vericiguat, is contraindicated because of the risk of excessive hypotension [5].

When combined with vasodilators or diuretics, monitoring of blood pressure and symptoms suggestive of orthostatic intolerance is recommended.

Overall, the pharmacological profile of vericiguat, characterised by predictable pharmacokinetics, limited interactions, and once-daily administration, may be particularly suitable for elderly patients with HF and polypharmacy. The principal randomised clinical trials evaluating vericiguat in HF are summarised in Table 3.

## 5. Clinical Evidence and Key Trials

### 5.1. The VICTORIA Trial

The pivotal Vericiguat Global Study in Subjects with Heart Failure with Reduced Ejection Fraction (VICTORIA) enrolled 5050 patients with chronic symptomatic HFrEF following a recent worsening event, defined as HHF or the need for intravenous diuretic therapy [16]. Participants were randomly assigned in a 1:1 ratio to vericiguat, up-titrated from 2.5 mg to 10 mg once daily, or placebo, in addition to GDMT. Median follow-up was 10.8 months.

The primary composite endpoint of CV death or first HHF occurred in 35.5% of patients receiving vericiguat and 38.5% of those assigned to placebo, corresponding to an absolute risk reduction of 3.0% (HR 0.90, 95% CI 0.82–0.98; *p* = 0.02). From a clinical perspective, this translates to an estimated number needed to treat of approximately 24 patients over 1 year to prevent one primary outcome event. Although the composite endpoint was significantly reduced, CV mortality alone did not differ significantly between treatment groups (HR 0.93, 95% CI 0.81–1.06), indicating that the overall treatment effect was largely attributable to fewer HHF. This distinction is particularly relevant when interpreting the role of vericiguat in patients with worsening HFrEF.

Subgroup analyses suggested that the treatment effect was generally consistent across age, sex, baseline ejection fraction, and diuretic-dose categories. Approximately 59% of participants were aged ≥ 65 years, while nearly 25% were aged ≥ 75 years, providing clinically relevant information regarding the use of vericiguat in elderly populations.

**Figure 4:**
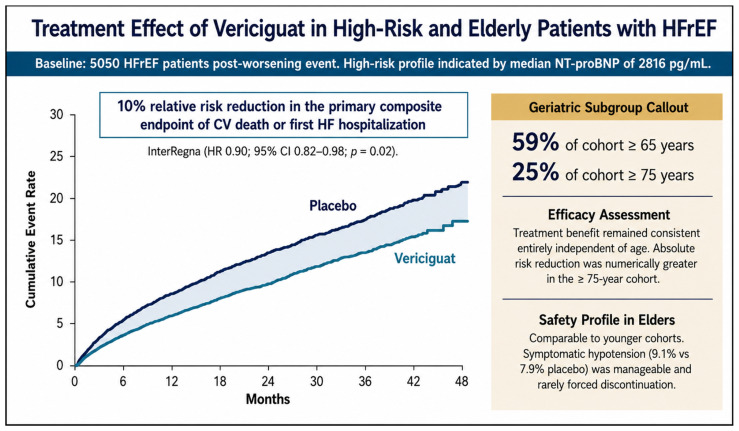
Effect of vericiguat on clinical outcomes in the VICTORIA trial. Illustrative representation of the treatment effect observed in the VICTORIA trial. The reduction in the primary composite outcome was mainly attributable to fewer HHF, whereas cardiovascular mortality was not significantly reduced. Age-related findings are derived from prespecified subgroup analyses and should be interpreted within the context of the overall trial, which was not specifically designed to evaluate frailty or other geriatric syndromes. Abbreviations: CI: confidence interval; CV: cardiovascular; HF: heart failure; HFrEF: heart failure with reduced ejection fraction; HR: hazard ratio; NT-proBNP: N-terminal pro-B-type natriuretic peptide.

Importantly, the safety profile observed in older participants was broadly comparable to that reported in younger patients. Symptomatic hypotension occurred in 9.1% of patients treated with vericiguat versus 7.9% in the placebo group, whereas syncope was reported in 4.0% and 3.5%, respectively. No significant increase in renal adverse events was observed, and haemoglobin reductions (mean −0.38 ± 1.27 g/dL) were modest and transient [35]. Additional analyses of the VICTORIA trial have shown that baseline NT-proBNP may also help identify patients most likely to benefit from vericiguat. Treatment effects appeared greatest in patients with moderately elevated NT-proBNP concentrations, whereas patients with extremely high baseline values showed less favourable outcomes, likely reflecting more advanced disease and greater clinical instability rather than reduced pharmacological efficacy [9,10,11]. Overall, these findings suggest that vericiguat may be considered in selected high-risk patients with HFrEF following recent worsening HF, particularly for reducing the burden of recurrent HHF (Figure 4).

### 5.2. Renal Function and Hemodynamic Sub-Analyses

Post hoc analyses from VICTORIA evaluated renal trajectories and baseline NT-proBNP levels. Median NT-proBNP concentration at enrolment was 2816 pg/mL, reflecting a population with advanced HF. The efficacy of vericiguat appeared generally consistent across eGFR categories, including patients with values as low as 15 mL/min/1.73 m^2^ [21,28]. Notably, no significant interaction between renal function and treatment effect was observed (*p* = 0.96), suggesting preserved benefit even in patients with moderate renal dysfunction, a common condition among older adults with HF.

The haemodynamic effects of vericiguat are thought to be primarily related to improvement in vascular compliance and ventricular–arterial coupling rather than direct natriuretic effects, consistentwith its cGMP-mediated mechanism of action.

### 5.3. SOCRATES-REDUCED

The SOCRATES-REDUCED trial was designed as a dose-finding and safety study in patients with chronic HFrEF and recent worsening HF [33]. Although the study did not meet its primary endpoint of a 12-week reduction in NT-proBNP, a dose–response relationship was observed, with greater reductions in the biomarker at higher vericiguat doses. Importantly, the trial confirmed acceptable tolerability, with rates of hypotension and syncope comparable to placebo. These findings contributed to the dose-escalation strategy subsequently adopted in the VICTORIA trial. Notably, SOCRATES-REDUCED included a substantial proportion of patients aged ≥ 70 years, among whom safety findings were similar to those observed in younger individuals, further supporting the feasibility of vericiguat use in elderly populations [33]. Although the primary endpoint was not achieved, higher vericiguat doses were associated with greater reductions in NT-proBNP, providing early evidence of biological activity on the NO–sGC–cGMP pathway and supporting the dose selection subsequently adopted in the VICTORIA trial [16]. Subsequent analyses of VICTORIA further demonstrated that vericiguat produced a sustained reduction in serial NT-proBNP concentrations compared with placebo, and that these changes were associated with improved clinical outcomes [9,10,11].

### 5.4. VITALITY-HFpEF and the NO–sGC Continuum

The VITALITY-HFpEF trial [4] evaluated vericiguat in 789 patients with HFpEF (ejection fraction ≥ 45%). Despite a strong pathophysiological rationale, treatment with vericiguat did not significantly improve Kansas City Cardiomyopathy Questionnaire (KCCQ) physical limitation scores or 6 min walk distance compared with placebo after 24 weeks of follow-up. Nevertheless, vericiguat was generally well tolerated, supporting the safety of cGMP pathway modulation in this clinical setting.

Collectively, these findings suggest that the clinical benefit of vericiguat is currently more evident in worsening HFrEF, whereas its role in HFpEF remains uncertain. However, modulation of the NO–sGC–cGMP pathway may still be relevant in selected HFpEF phenotypes characterised by endothelial dysfunction and vascular stiffness, especially in elderly and clinically complex patients. Therefore, current evidence does not support the routine use of vericiguat in older patients with HFpEF.

### 5.5. The VICTOR Trial

The recently published VICTOR trial [18,19] evaluated the efficacy and safety of vericiguat in 6105 ambulatory patients with chronic HFrEF without recent worsening HF, thereby extending the evidence base beyond the high-risk population enrolled in VICTORIA [16]. Participants had a left ventricular ejection fraction ≤ 40% and were receiving contemporary guideline-directed medical therapy, including widespread use of ARNI and SGLT2 inhibitors. Unlike VICTORIA, which focused on patients following a recent worsening of HF, VICTOR investigated a more stable ambulatory HFrEF population [34]. Vericiguat did not significantly reduce the primary composite endpoint of CV death or HHF compared with placebo. However, despite the neutral primary endpoint, lower rates of CV and all-cause mortality were observed in the vericiguat group. These findings should be interpreted within the context of the overall trial results and provide complementary information regarding the potential role of vericiguat across different stages of HFrEF severity. These findings provide important complementary information regarding the potential role of vericiguat across different stages of HFrEF severity and support further exploration of its use in contemporary HF management. Furthermore, a prespecified individual participant-level pooled analysis of the VICTORIA and VICTOR trials, including 11,155 patients, demonstrated significant reductions in the composite endpoint of CV death or HHF, as well as in CV mortality, further supporting the role of vericiguat across a broad spectrum of HFrEF severity.

### 5.6. Integration with Guidelines

According to the 2023 ESC Focused Update and the 2022 AHA/ACC/HFSA Guidelines, vericiguat may be considered in patients with symptomatic HFrEF (ejection fraction ≤ 40%) who experience worsening HF despite optimised GDMT [29,30]. Its positioning within the therapeutic algorithm generally follows initiation of ARNI and SGLT2i, particularly in patients at high risk of recurrent HHF. Importantly, the available evidence suggests that vericiguat is best positioned in patients with symptomatic worsening HFrEF despite optimised GDMT, particularly following a recent worsening HF event. Baseline NT-proBNP may further contribute to patient selection, as current evidence suggests greater benefit in patients with elevated, but not extremely high, NT-proBNP concentrations [9,10,11].

Given its relatively neutral effects on heart rate, serum potassium, and renal function, vericiguat may represent a reasonable therapeutic option in selected older adults with limited tolerance to further up-titration of conventional neurohormonal therapies [1,36].

## 6. Vericiguat in Elderly and Frail Patients

**Figure 5:**
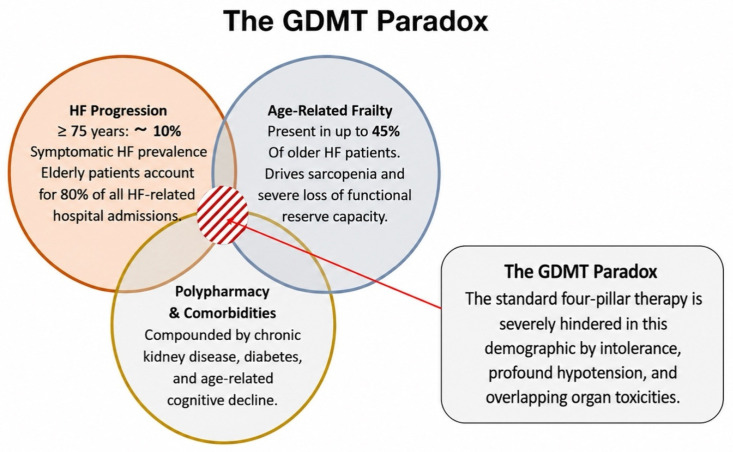
The vulnerability intersection in elderly heart failure.

Older adults with HF represent a clinically heterogeneous and physiologically vulnerable population requiring tailored therapeutic strategies that account for frailty, multimorbidity, and altered pharmacodynamic responses (Figure 5). Frailty affects up to 45% of elderly patients with HF and is independently associated with recurrent HHF, functional decline, and mortality. The interaction between sarcopenia, chronic inflammation, autonomic dysfunction, and reduced physiological reserve may also influence tolerance and response to CV therapies.

In this context, vericiguat may represent a potentially useful therapeutic option for several reasons. First, it acts through the NO–sGC–cGMP pathway, which remains active independently of β-adrenergic or renin–angiotensin system blockade, thereby avoiding excessive cumulative neurohormonal inhibition. Second, its once-daily administration and relatively favourable safety profile may facilitate adherence in patients exposed to polypharmacy. Third, post hoc analyses from the VICTORIA trial suggested consistent efficacy across age categories, with numerically greater absolute risk reduction among patients aged ≥ 75 years, likely reflecting their higher baseline event risk [16,35].

In addition, no clinically significant pharmacokinetic interactions have been reported with ARNI, MRA, or SGLT2i, which are frequently co-prescribed in older adults with HF.

Frailty commonly coexists with CKD, anaemia, cognitive impairment, and functional limitation, conditions that frequently hinder optimal up-titration of β-blockers or renin–angiotensin–aldosterone system inhibitors. In these settings, the relatively neutral renal and metabolic profile of vericiguatmay offer a potential adjunctive therapeutic option. In the VICTORIA trial, the modest reduction in haemoglobin levels (mean −0.38 g/dL) appeared transient and clinically limited [35].

Similarly, although symptomatic hypotension was slightly more frequent than with placebo, treatment discontinuation remained uncommon.

Taken together, these findings suggest that vericiguat may be considered as part of an individualized treatment strategyas part of an individualised treatment strategy when full implementation of conventional neurohormonal therapies is not feasible. Beyond pharmacological considerations, initiation of vericiguat in frail older adults should ideally incorporate multidimensional geriatric assessment tools, including the Clinical Frailty Scale, gait speed evaluation, and Barthel Index, in order to identify patients most likely to benefit from therapy. Shared decision-making among patients, caregivers, cardiologists, and primary care physicians remains essential for aligning treatment intensity with functional status and quality-of-life priorities (Table 4). Although VICTORIA [16] included a substantial proportion of older adults, including more than 1500 participants aged ≥ 75 years, the trial was not specifically designed to evaluate geriatric syndromes such as frailty, cognitive impairment, institutionalisation, or severe polypharmacy. Therefore, evidence in these highly vulnerable subgroups remains limited and further dedicated studies are warranted. Until such evidence becomes available, treatment decisions should be individualised and guided by a comprehensive clinical and geriatric assessment.

Conceptual illustration of the interplay among ageing-related frailty, HF progression, and multimorbidity in older adults. These factors may interact to increase clinical vulnerability, thereby increasing the risk of HHF and potentially limiting the implementation or optimisation of guideline-directed medical therapy. The figure summarises the current conceptual understanding based on the available literature and should not be interpreted as direct evidence of causal relationships or specific therapeutic recommendations.

### Vericiguat in Elderly Patients with HFpEF

The age structure of both HFpEF trials deserves closer scrutiny. In VITALITY-HFpEF, the mean age of the enrolled population was 72.7 years, meaning that the neutral result on the KCCQ physical limitation score was obtained in a cohort already dominated by older adults rather than in a younger reference group extrapolated to the elderly [4]. Similarly, SOCRATES-PRESERVED enrolled patients with a mean age of 73 years, and it was precisely in this older cohort that the 10 mg dose produced a clinically meaningful gain in the KCCQ clinical summary score despite no measurable change in NT-proBNP or left atrial volume [37]. Taken together, these two studies indicate that age alone did not preclude a signal of benefit on patient-reported outcomes, yet the larger and longer VITALITY-HFpEF trial was unable to reproduce it, a discrepancy that has been attributed to a smaller separation from placebo, a less symptomatic baseline population, and possibly the play of chance in the earlier phase II study [4,38]. Neither trial, however, incorporated a frailty index, a comprehensive geriatric assessment, or a prespecified subgroup analysis restricted to the oldest or most comorbid patients, so it remains unclear whether the mechanism targeted by vericiguat behaves differently across gradients of biological rather than chronological age. As recently summarised in a broader review of vericiguat’s pharmacology and clinical evidence, the drug’s favourable tolerability profile in HFrEF has not been matched by a comparable efficacy signal in HFpEF, and the authors explicitly call for further work to clarify its role across the ejection fraction spectrum before any age-specific recommendation can be made [39]. Until such data become available, the use of vericiguat in older patients with HFpEF should continue to be guided by the overall neutral efficacy findings from VITALITY-HFpEF rather than by the more favourable but preliminary quality-of-life signal from SOCRATES-PRESERVED.

## 7. Practical Considerations for Clinical Implementation

### 7.1. Initiation and Titration

**Figure 6:**
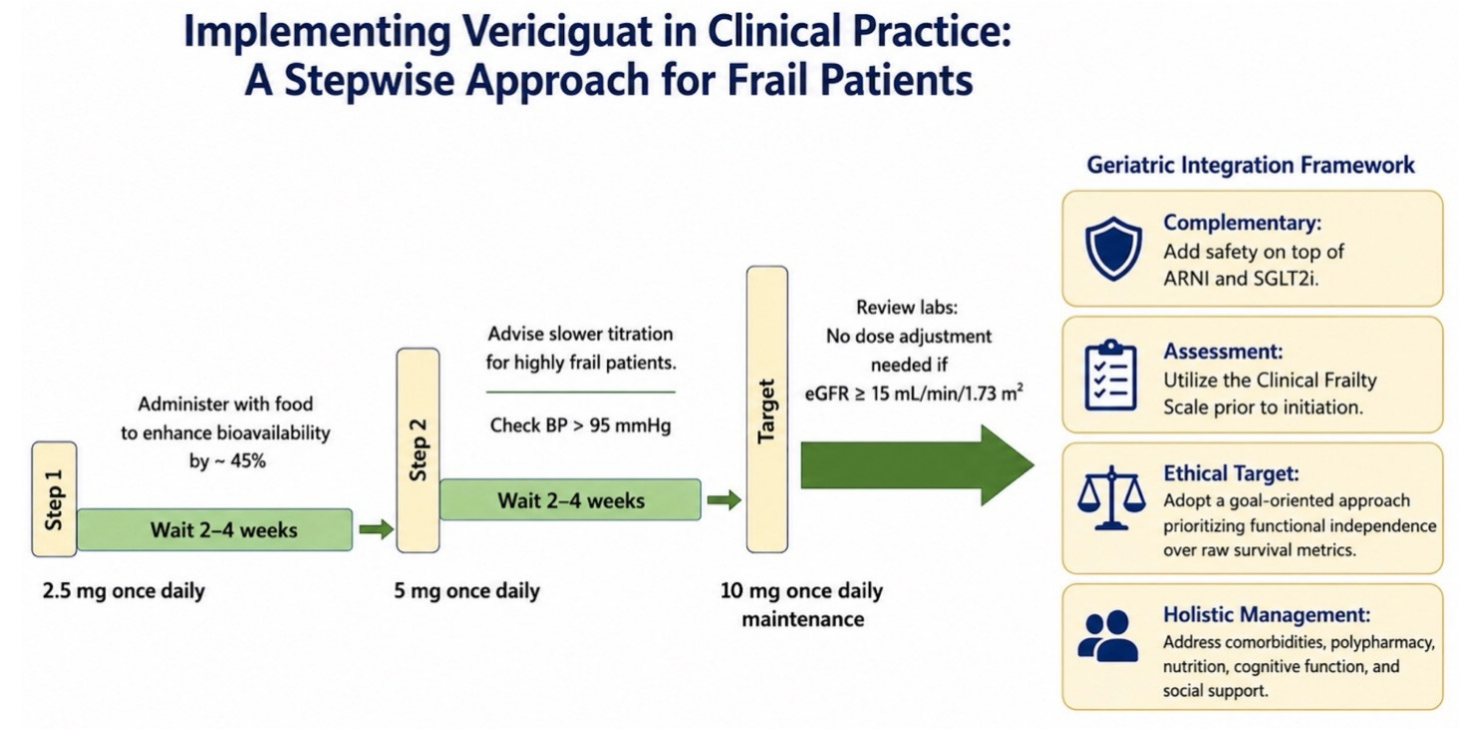
Stepwise implementation of vericiguat in frail patients with heart failure.

Vericiguat should be initiated in clinically stable patients with HFrEF who have recently experienced worsening HF despite GDMT. The recommended starting dose is 2.5 mg once daily administered with food, with progressive up-titration every two weeks (2.5 mg → 5 mg → 10 mg) according to clinical tolerability until the target dose of 10 mg is achieved [40] (Figure 6). Food intake increases vericiguat bioavailability by approximately 45%. In elderly or hypotensive patients, a slower titration strategy, with dose escalation every 3–4 weeks, may be more appropriate. Blood pressure should be assessed at baseline and reassessed during the first weeks after treatment initiation. In most cases, therapy can be maintained if systolic blood pressure remains > 95 mmHg. Renal function and haemoglobin levels should also be periodically monitored during titration. No dose adjustment is generally required in patients with eGFR ≥ 15 mL/min/1.73 m^2^ or mild-to-moderate hepatic impairment, although vericiguat has not been adequately evaluated in patients undergoing dialysis.

Recommended titration strategy starting from 2.5 mg once daily and progressively increasing to the target dose of 10 mg with monitoring of blood pressure, renal function, and clinical tolerance.

### 7.2. Combination with Contemporary Therapy

In the era of contemporary quadruple HF therapy, vericiguat should be considered as a complementary rather than a substitutive treatment option. In the VICTORIA trial [16], approximately 60% of patients were receiving β-blockers, 70% were receiving ACE inhibitors or angiotensin receptor blockers (ACEI/ARBs), 15% were receiving ARNI, and 73% were receiving MRA [16]. Subsequent analyses suggested that the effects of vericiguat were generally maintained among patients already receiving ARNI and SGLT2i [34].

From a mechanistic perspective, cGMP-mediated vasorelaxation and improved ventricular–arterial coupling may complement the haemodynamic and metabolic effects of SGLT2i and ARNI therapy. Because vericiguat does not significantly affect heart rate, serum potassium, or cardiac conduction, it may represent a reasonable therapeutic option in selected patients with conduction disturbances or borderline potassium levels.

Polypharmacy nevertheless remains a major challenge in elderly patients with HF. Consequently, periodic medication review and careful therapeutic reconciliation are advisable to minimise the risk of excessive vasodilation, symptomatic hypotension, and potentially inappropriate drug combinations.

### 7.3. Potential Risks and Practical Considerations in Elderly Patients

Despite its overall favourable safety profile, prescribing vericiguat in older adults calls for careful individual judgement, since age-related physiological decline, multiple coexisting conditions, and extensive concurrent medication use can all shape both how well the drug is tolerated and how patients respond to it [40]. Symptomatic hypotension and syncope were reported only infrequently across the pivotal trials, yet older patients are more prone to blunted autonomic reflexes and wider swings in blood pressure, which can make them more vulnerable to these effects than younger trial participants [4,16]. Checking baseline blood pressure, asking about orthostatic symptoms, and re-evaluating patients periodically during up-titration are therefore sensible precautions, particularly when other vasodilating agents or high-dose diuretics are being used concomitantly. Impaired kidney function is another frequent feature of the older heart failure population. Data from the VICTORIA trial indicate that vericiguat’s effect on renal parameters is largely neutral, with no consistent signal of clinically important worsening in kidney function [40]. Even so, periodic monitoring of renal parameters remains prudent, particularly after episodes of decompensation or when other treatments affecting circulating volume or renal perfusion are adjusted. A mild decline in haemoglobin has also been documented during treatment, supporting intermittent laboratory monitoring in older patients with anaemia or multiple coexisting illnesses [4,35]. From a pharmacological standpoint, vericiguat. has been considered to carry a low risk of clinically significant drug interactions, a genuine advantage given that older patients often take numerous CV and non-CV medications [41]. That said, deciding whether and how to use the drug cannot rest on its pharmacokinetic profile alone. A thorough review of the patient’s full medication list, paired with an individualised look at frailty, functional and cognitive status, and personal treatment goals, remains indispensable for tailoring therapy and keeping the overall medication burden in check. Vericiguat should therefore be regarded as one component of a broader, patient-centred care plan rather than as a stand-alone pharmacological fix [24].

### 7.4. Health-Economic and Ethical Aspects

From a health care system perspective, elderly patients with HF account for a substantial proportion of recurrent HHF and cumulative health-related costs. In the VICTORIA trial, vericiguat was associated with a relative reduction in the composite endpoint of CV death or HHF (HR 0.90; 95% CI 0.82–0.98) [16]. Although dedicated cost-effectiveness analyses in elderly populations remain limited, a reduction in recurrent HF events may potentially translate into lower health care utilisation, particularly in universal health care systems such as the Italian National Health Service.

Nevertheless, the incremental clinical benefit of vericiguat should be interpreted within the broader context of biological age, frailty burden, comorbidity profile, life expectancy, and patient-centred therapeutic goals. Therapeutic appropriateness should therefore avoid age-based discrimination while simultaneously considering functional status, expected quality-of-life benefit, and treatment sustainability.

From an ethical perspective, the management of elderly patients with HF should ideally follow a goal-oriented, patient-centred approach focused on preserving autonomy, functional capacity, and quality of life, rather than exclusively on prolonging survival. Shared discussions regarding expected benefit, pill burden, adherence feasibility, and patient preferences remain essential components of therapeutic decision-making in frail older adults.

## 8. Future Directions

Although vericiguat expands the therapeutic landscape for HFrEF, several important areas remain underexplored, particularly in elderly and frail populations.

First, evidence specifically focused on frailty remains limited. RCTs rarely incorporate multidimensional geriatric assessment, cognitive evaluation, or functional status measures, despite the major influence of these variables on adherence, tolerability, and clinical outcomes. Dedicated studies enrolling patients aged ≥ 80 years are needed to better define the long-term safety, tolerability, and effectiveness of vericiguat in very old and clinically vulnerable populations.

Second, biomarker-guided therapeutic strategies deserve further investigation. Post hoc analyses from VICTORIA suggested an attenuation of treatment benefit in patients with NT-proBNP concentrations > 8000 pg/mL [23]. Identification of more precise biomarker thresholds may improve patient selection and therapeutic timing. In addition, integration of biomarkers reflecting endothelial dysfunction, cGMP signalling, or vascular inflammation could potentially contribute to a more personalised treatment approach.

Third, comparative effectiveness studies evaluating vericiguat alongside contemporary HF therapies, including SGLT2i and emerging agents such as omecamtivmecarbil, may help clarify optimal sequencing and integration strategies. Given their potentially complementary mechanisms of action, combined therapeutic approaches may prove beneficial in selected high-risk elderly patients with advanced HFrEF.

Fourth, implementation science and real-world registries may provide important information regarding prescription patterns, adherence, tolerability, and health care utilisation in geriatric HF populations. Integration of telemedicine and remote monitoring strategies could facilitate safer titration and closer follow-up, particularly in home-bound, frail, or cognitively impaired patients. Early experiences from multidisciplinary HF programmes suggest that virtual follow-up may improve adherence and facilitate early identification of treatment-related adverse effects.

Finally, further translational research is warranted to better understand the potential effects of vericiguat on myocardial energetics, mitochondrial function, endothelial dysfunction, and vascular ageing, mechanisms that may be particularly relevant in elderly patients with HF [16].

## 9. Limitations

This review has some limitations that should be acknowledged. Although the literature search followed predefined eligibility criteria and study selection was summarised using a PRISMA-style flow diagram to improve transparency, the review was conceived as a structured narrative review rather than a formal systematic review or meta-analysis. For this reason, no formal assessment of study quality, risk of bias, or certainty of evidence was performed. The findings presented here should therefore be viewed as a qualitative overview of the available literature rather than as evidence generated through a formal systematic review.

Another limitation is the limited amount of evidence specifically addressing older adults with HF. While the VICTORIA trial included a substantial proportion of elderly patients, dedicated RCTstudies focusing on frailty, multimorbidity, cognitive impairment, and other geriatric syndromes remain scarce. As a result, several considerations discussed in this review rely on subgroup analyses, observational studies, and real-world experience, each of which has inherent methodological limitations.

Finally, evidence on vericiguat continues to expand. Recently published RCTS, pooled analyses, and real-world studies have further refined its clinical positioning, but additional research is still needed to better define its role in frail older patients and other underrepresented populations.

## 10. Conclusions

Current evidence suggests that vericiguat may representan additional therapeutic option within the contemporary management of worsening HFrEF. By targeting the impaired NO–sGC–cGMP pathway, which contributes to endothelial dysfunction and oxidative stress in HF, vericiguat acts through a mechanism complementary to conventional neurohormonal therapies [4,16,33]. This benefit was largely attributable to fewer HHF, whereas CV mortality was not significantly reduced, while the overall tolerability profile remained favourable across different age groups.

In elderly and frail patients, vericiguat may be particularly relevant because of its once-daily administration, limited drug–drug interactions, and relatively neutral effects on renal function, potassium balance, and heart rate. Nevertheless, therapeutic decisions in older adults should remain individualised and integrated within a multidimensional geriatric-cardiology framework that incorporates frailty assessment, comorbidity burden, shared decision-making, and quality-of-life priorities.

While additional evidence from future real-world studies and dedicated investigations in vulnerable older populations is awaited, the available evidence suggests that vericiguat may be considered a reasonable adjunctive option in selected elderly patients with HFrEF, particularly following worsening HF despite GDMT. Recent evidence from the VICTOR trial and the subsequent pooled analysis with VICTORIA has expanded the evidence base beyond patients with recent worsening heart failure, suggesting potential benefit across a broader spectrum of HFrEF. NT-proBNP may also contribute to identifying patients most likely to benefit from therapy, although this strategy requires further prospective validation before being routinely incorporated into treatment algorithms. Nevertheless, the available evidence is currently strongest for patients with HFrEF, whereas studies in HFpEF have not demonstrated significant clinical benefit and do not support routine use in this population.

## Figures and Tables

**Table 1:** Clinical studies evaluating vericiguat in older patients with heart failure.

Study	Year	Design	Population	Mean Age/ Elderly Subgroup	Follow-Up	Main Efficacy Findings	Safety Findings	Relevance for Older Adults
VICTORIA (Armstrong et al.) [16]	2020	Phase III multicenter RCT	5050 patients with worsening chronic HFrEF (LVEF < 45%) after recent HF decompensation	Mean age 67.3 years	10.8 months	Vericiguat reduced composite CV death/HF hospitalization (HR 0.90)	Similar adverse event rates vs placebo; modest hypotension/syncope	Foundational trial; elderly represented but not specifically frailty-focused
VICTORIA age/sex analysis Lam et al. [17]	2023	Post hoc subgroup analysis	VICTORIA cohort	Older subgroup analysis	As VICTORIA	Treatment benefit preserved across age strata	Acceptable tolerability in older participants	Direct evidence supporting efficacy irrespective of age
VICTOR trial [18,19]	2025	Phase III RCT	Stable chronic symptomatic HFrEF without recent worsening HF	Older adults included	Median 18.5 months	Primary endpoint not significantly reduced; lower CV and all-cause mortality observed	Favorable safety profile; slightly higher rates of symptomatic hypotension	Contemporary applicability in optimally treated ambulatory HFrEF
Zannad et al. pooled IPD analysis [20]	2025	Prespecified individual participant-level pooled analysis	VICTORIA + VICTOR (11,155 patients)	Broad age spectrum	Extended	Reduced CV death/HF hospitalization, CV mortality and HF hospitalization across the HFrEF risk spectrum	Safety maintained across studies	Strong age-relevant evidence across a broad range of HFrEF severity
German nationwide cohort [21]	2024	Real-world observational cohort	2916 incident vericiguat users	Mean age ~73 years	Real-world follow-up	Good adherence; used in high-risk HF population	Lower titration success in older adults	Excellent elderly real-world applicability
VERISEC Registry (Esteban-Fernández et al.) [22]	2024	Prospective multicentre real-world registry	776 patients treated with vericiguat in routine clinical practice	Mean age 72.4 years; high comorbidity burden	Cross-sectional analysis	Patients older and clinically more complex than VICTORIA population	Safety profile consistent with routine clinical use	Strong real-world evidence in elderly, multimorbid HF patients
VERITA study [23]	2026	Prospective observational	103 HFrEF patients post worsening HF	Older/high-risk cohort	Mid-term	Improved NYHA class, QoL, fewer urgent HF visits	Low discontinuation	Practical post-discharge elderly relevance
Armentaro et al. geriatric cohort [24]	2026	Prospective observational	55 elderly HFrEF patients	Explicit elderly cohort	Short/intermediate	Improvements in NT-proBNP, LVEF, gait speed, cognition	Well tolerated	Direct geriatric evidence
Ma et al. meta-analysis [25]	2023	Meta-analysis	RCTs	Mixed populations	Variable	Reduced composite HF events	Neutral mortality effect	Indirect supportive evidence
Darmadi et al. systematic review/meta-analysis [26]	2025	Systematic review	HF studies	Mixed	Variable	More conservative pooled efficacy	Safety acceptable	Contextual evidence
Spadafora et al. review [27]	2024	Narrative review	Older complex HF patients	Elderly focus	—	Positioning of vericiguat in complex elderly HF	Discusses hypotension/polypharmacy	Strong conceptual relevance
Polypharmacy meta-analysis (Akbar et al.) [28]	2025	Meta-analysis	Elderly HF	Elderly	Variable	Polypharmacy linked to worse outcomes	Medication burden concerns	Supports rationale for vericiguat simplification strategy

**Table 2:** Pathophysiological Basis of Vericiguat in Heart Failure.

Mechanism	Physiological Effect	Clinical Implication
Direct sGC stimulation	Restores cGMP synthesis independently of nitric oxide availability	Counteracts oxidative stress-mediated endothelial dysfunction
sGC sensitization to endogenous NO	Enhances vasodilation and myocardial relaxation	Improves diastolic filling and microvascular perfusion
Anti-fibrotic signaling via PKG	Reduces myocardial and vascular fibrosis	Limits adverse remodeling and improves ventricular compliance
Improved mitochondrial function	Enhances cellular energetics and reduces apoptosis	Supports myocardial contractility in the aging heart

Abbreviations: sGC: soluble guanylate cyclase; cGMP: cyclic guanosine monophosphate; NO: nitric oxide; PKG: protein kinase G.

**Table 3:** Key Clinical Trials of Vericiguat in Heart Failure.

Trial	Population (*n*)	Design/EF (%)	Main Findings	Notes in Elderly
SOCRATES-REDUCED [33]	456	HFrEF < 45	NT-proBNP ↓ (dose–response); safe	Included ≥ 70 yrs, good tolerability
VICTORIA [16]	5050	HFrEF < 45	↓ CV death/HF hosp HR 0.90 [0.82–0.98] *p* = 0.02	59% ≥ 65 yrs; consistent benefit
VITALITY-HFpEF [4]	789	HFpEF ≥ 45	No KCCQ gain; safe	Demonstratedsafety in olderadults
VICTOR [19,34]	6105	Stable chronic HFrEF, LVEF ≤ 40%	Primary endpoint (CV death or HF hospitalization) not significantly reduced; lower cardiovascular and all-cause mortality observed	Broad representation of older adults; contemporary GDMT including widespread ARNI and SGLT2i use

HF: heart failure; HFrEF: heart failure with reduced ejection fraction; HFpEF: heart failure with preserved ejection fraction; EF: ejection fraction; NT-proBNP: N-terminal pro-B-type natriuretic peptide; CV: cardiovascular; HR: hazard ratio; KCCQ: Kansas City Cardiomyopathy Questionnaire; ARNI: angiotensin receptor-neprilysininhibitor; SGLT2i: sodium-glucose cotransporter-2 inhibitor.

**Table 4:** Practical Use of Vericiguat in Elderly Patients.

Clinical	Suggested Strategy	Monitoring	Comments
Post-HF decompensation	Start 2.5 mg → 10 mg with food	BP every 2–4 weeks	Check for symptomatic hypotension
Frail/polymedicated	Slow titration; avoid overlap with vasodilators	Review medications	Low interaction risk
CKD (eGFR 15–30)	No dose change; monitor Cr/Hb	Labs monthly first 3 mo	Stable renalprofile
Cognitive impairment	Simplify regimen; involve caregivers	Adherence checks	Once-dailydosingimproves compliance

Abbreviations: HF: heart failure; BP: blood pressure; CKD: chronic kidney disease; eGFR: estimated glomerular filtration rate; Cr: creatinine; Hb: haemoglobin; mo: months.

## Data Availability

No new data were created or analyzed in this study. Data sharing is not applicable to this article.
